# Strength and Microstructure of Class-C Fly Ash and GGBS Blend Geopolymer Activated in NaOH & NaOH + Na_2_SiO_3_

**DOI:** 10.3390/ma13010059

**Published:** 2019-12-20

**Authors:** Sasui Sasui, Gyuyong Kim, Jeongsoo Nam, Tomoyuki Koyama, Sant Chansomsak

**Affiliations:** 1 Department of Architectural Engineering, Chungnam National University, Daejeon 34134, Korea; sassuikhuwaja126@gmail.com (S.S.); j.nam@cnu.ac.kr (J.N.); 2 Department of Architecture and Urban Design, Kyushu University, Fukuaka 812-0053, Japan; koyama@arch.kyushu-u.ac.jp; 3 Department of Architecture, Naresuan University, Phitsanulok 65000, Thailand; santc@nu.ac.th

**Keywords:** geopolymer, fly ash, GGBS, NaOH, NaOH + Na_2_SiO_3_, strength, microstructure

## Abstract

In this paper, class-C fly ash (FA) and ground granulated blast-furnace slag (GGBS)-based geopolymer activated in NaOH and NaOH + Na_2_SiO_3_ was studied regarding setting time, compressive strength, porosity, microstructure, and formation of crystalline phases. When comparing the effects of alkali type on the FA and GGBS geopolymer composites, results revealed that NaOH has a lesser effect in developing strength and denser microstructure than does NaOH + Na_2_SiO_3,_ since the addition of Na_2_SiO_3_ provides the silica source to develop more compact structure. Incorporation of Na_2_SiO_3_ reduced the crystallinity and the paste was more amorphous compared to NaOH activated pastes. The class-C FA and GGBS blends resulted in prolonged setting time, reduced strength, and loose matrix with the increase in fly ash content. The un-reactivity of calcium in blends was observed with increasing fly ash content, leading to strength loss. It is evident from XRD patterns that calcium in fly ash did not contribute in forming C-S-H bond, but formation of crystalline calcite was observed. Furthermore, XRD analyses revealed that the reduction in fly ash leads to the reduction in crystallinity, and SEM micrographs showed the unreactive fly ash particles, which hinder the formation of a denser matrix.

## 1. Introduction

Geopolymer cement can be one of the alternatives to replace the use of Portland cement in pre-cast and non-structural applications as it utilizes the industrial by-product waste, due to which, the occupancy of landfills reduces and prevents the ecology and environment from harmful effects [1,2]. Additionally, the replacement of Portland cement reduces the emission of greenhouse gases as well as the consumption of excessive energy [3].

Generally, the synthesis of geopolymer cement involves the activation between the alumino silicate material and alkali solution. During the polymerization, the formation of bonds depends on the chemical composition of the base material and the type of alkali solution. For example, the activation of fly ash generally forms –Si-O-Si- or –Si-O-Al- bonds depending on the Si-Al ratio, which varies with the chemical composition of the precursor material and alkali solution [4,5,6,7]. The formation of C-S-H, C-A-H, or C-A-S-H bond is observed when high-calcium materials such as slag are activated in the alkali solution. It allows Al^3+^ and Si^4+^ to react with Ca^2+^ during polymerization [8]. This type of bond is also formed in Portland cement. Its formation leads to short setting time, reduced porosity, and increased compressive strength [9,10].

Fly ash has been investigated as a precursor material in geopolymer mortar. The mechanical properties of fly ash-based geopolymer depends on the chemical composition of fly ash. As explored by Davidovits, Izquierdo [11] in their study, the fly ash containing low calcium (class-F) produced a workable geopolymer; however, the high calcium in fly ash did not contribute in producing the suitable geopolymer due to flash setting time. The flash setting time in the high-calcium fly ash was confirmed in studies [12,13] where they used borax in the mixture to delay the setting time. However, regardless of this problem, the class-C fly ash has been found to help improve the strength properties due the presence of adequate calcium content, which may forms C-S-H gel in the matrix [14].

Ground granulated blast-furnace slag (GGBS) is another by-product material rich in amorphous calcium, silica, and alumina, which makes it suitable to be used as a binder in the construction industry [15]. Formerly, GGBS as a precursor material in geopolymer has been investigated. It is worth mentioning that the GGBS activated in alkali solution possess a high strength and good resistance to the chemicals compared to Portland cement. However, the reduced setting time makes it susceptible to high shrinkage and develops micro cracks, which increases the porosity leading to the strength loss [16]. Since the GGBS is rich in calcium, the main reaction product is C-S-H gel, which may coexist with the geopolymer gel depending on the chemical composition of the GGBS, alkali type, and alkali amount [4,17,18].

The combined effects of low-calcium fly ash (class-F) and GGBS on the strength development and microstructure of geopolymer mortar have been explored by many studies. As revealed by Wang, Wang [19] and Qiu, Zhao [20] in their study, the strength increases and the matrix becomes more homogeneous as the ratio of GGBS to fly ash increases due to the increase in the calcium content, leading to the formation of C-S-H and C-A-S-H bonding. Puertas, Martínez-Ramírez [21] further revealed in their study that the GGBS in the composite mix is more reactive than is low-calcium fly ash, which contributes to the final strength. On the contrary, Chi and Huang [22], Wardhono, Law [23] and Ling, Wang [24] revealed that the increasing amount of GGBS in a mix (i.e., above 50% by wt.) reduced the strength. The reasons attributed are the development of micro-cracks due to the excessive calcium in GGBS, the occurrence of expansion, and cracks due to the development of Ca(OH)_2_ in the presence of excessive calcium [25]. However, the research on low-calcium fly ash and slag composite is conflicting in terms of GGBS to fly ash ratio in developing strength and microstructure of the composite.

Based on the reviews, it can be attributed that the excessive calcium content may lead to the decreased strength. Due to this fact, the composite of high calcium GGBS and high calcium fly ash have been less explored with only the work of Chen, Diaz, Menozzi and Murillo [26]. The investigations in their work are limited as the composites are investigated with respect to strength, while the microstructure and elemental properties were not determined. The study on the types of alkali activators, i.e., NaOH and Na_2_SiO_3_ for composite class-C fly ash and GGBS is scarce. Therefore, this paper investigated and reports the combined effects of class-C fly ash and GGBS at different mass ratios synthesized in two different alkali solutions i.e., NaOH and NaOH + Na_2_SiO_3_. Where, the focus was to explore the contribution of the composites at different ratios and alkali activators on the setting time, strength, porosity, crystallinity and microstructure of the geopolymer composites. The obtained results would benefit the future applications of composite class-C fly ash and GGBS.

## 2. Experimental Plan

### 2.1. Materials

**Figure 1:**
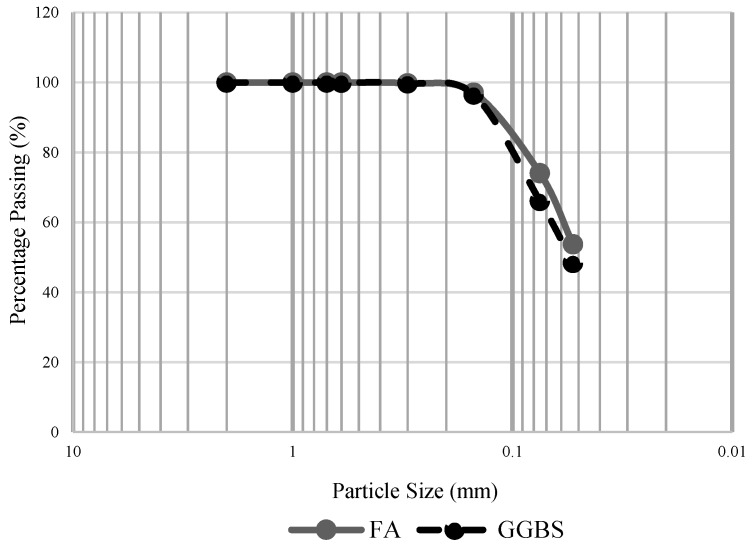
Particle size distribution of FA and GGBS.

In this study, the class-C fly ash (FA) as per ASTM C618-19 and ground granulated blast-furnace slag (GGBS) was used as a raw material. The elemental and oxide compositions obtained from SEM-EDS (model LYRA3 XMU) are shown in Table 1. Figure 1 shows the particle size distribution of FA and GGBS obtained by sieving, indicating that the size of about half of the fly ash and GGBS particles was lower than 53 µm. To activate the raw materials, two alkali solutions were used, i.e., sodium hydroxide (NaOH) and combined sodium hydroxide and sodium silicate (NaOH + Na_2_SiO_3_). The NaOH solution was prepared by dissolving NaOH pellets (assay 97%) in distilled water to obtain a concentration of 4 M. It is optimal to use this considering the hardening of high-calcium composites, i.e., class-C FA and GGBS [27,28,29]. Additionally, at low hydroxyl concentration, the dissolving of Ca^2+^ increases, leading to the maximum formation of C-S-H in the matrix [30]. The prepared solution was cooled to room temperature for 24 h before use. To prepare the NaOH + Na_2_SiO_3_, the synthetically produced Na_2_SiO_3_ (molecular weight 122.06; assay: 9%–10% Na_2_O, 28%–30% SiO_2_) was used and mixed with a freshly made NaOH (4M) solution then cooled to room temperature for 24 h. The optimal ratio of Na_2_SiO_3_/NaOH = 1.5 was used in this study to avoid the excessive silicates in mixture, which may negatively affect the geopolymeric structure formation, as well as water evaporation [31,32].

### 2.2. Sample Preparation

The composite of FA and GGBS was mixed at a mixing ratio set by weight percentage as shown in Table 2. Each dry mixture was then activated in two different alkali solutions, i.e., NaOH (Cem.C) and NaOH + Na_2_SiO_3_ (CemN.C) at a constant alkali/solid material (Al/S) ratio of 0.4. To cast the specimens, the dry mix was mixed in a Hobart mixer at low speed for 10 min to ensure the materials were combined well. The alkali solution was then added to the mixer where both materials were mixed at a moderate speed for five minutes. Water was gradually added in an amount required by each mixture to achieve the same consistency, i.e., same slump measured as recommended by Tan, Bernal [33]. Table 2 shows the ratio of water to alkali added paste (W/Mix), added in each mixture, it can be clearly seen that, with the increasing amount of GGBS, the demand of water increased. The increased water requirement with increasing GGBS was due to its chemical reactivity, and specific gravity which was comparatively higher than FA [34,35]. Comparatively, the water demand for all CemN.C pastes increased compared to Cem.C paste due to the increased viscosity of the solution when Na_2_SiO_3_ was added in NaOH.

After adding water, the paste was mixed for an additional 15 min to obtain the homogeneous mixture. The paste was then poured in metallic molds in two layers while vibrated mechanically on the vibrating compactor for 2.5 min, then sealed immediately with plastic wrap to prevent the loss of moisture. The molds were kept at 22 °C and 45% relative humidity for two hours before it was cured in an oven at 60 °C for 24 h to prevent the quick evaporation of moisture from the paste. After the oven cure, the molds were unsealed and rested for two hours in indoor laboratory condition to reduce the mold’s temperature. The specimens were then removed from the molds and cured for 28 days in a laboratory condition, where temperature was 23 (±5) °C and relative humidity was 55 (±5)%.

### 2.3. Test Methods

Fresh mortar was used to determine the setting time in a laboratory condition. The test was conducted in accordance to ASTM C191-19 and data was collected at an interval of five minutes. Cubic specimens of 50 mm^3^ were used to determine the compressive strength in accordance to ASTM C109/C109M-16a. The Archimedes principle was applied to the geopolymer specimens as per the procedure described in the ASTM C20-00 standard to determine the physical properties, i.e., volume of impervious portion, apparent porosity, and water absorption. The samples after the compression test were collected and then ground to form powder for the XRD analyses, where the powder XRD was performed at 2θ from range for 5° to 100° using X-ray Diffractometer (D/Max-2200 Ultima/PC). Part of the collected samples were Pt. coated for SEM and EDS analysis which was carried out in FIB-SEM, model LYRA3 XMU to determine the major elements in the paste and to observe the microstructure.

## 3. Results and Discussions

### 3.1. Final Setting Time

**Figure 2:**
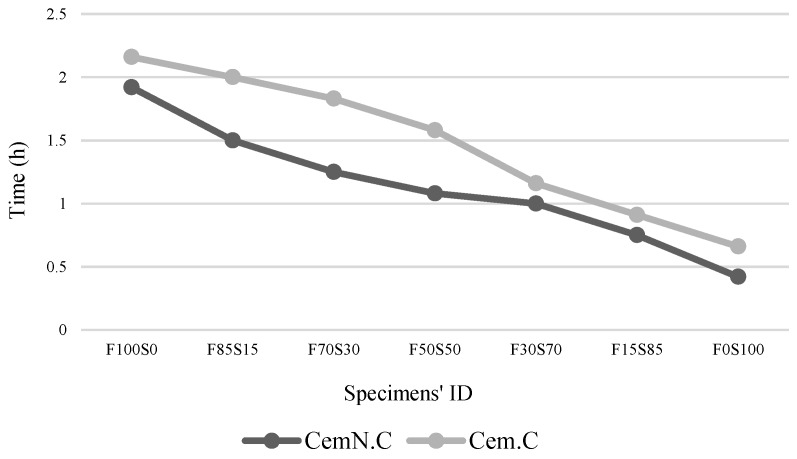
Final setting time of Cem.C and CemN.C activated FA/GGBS pastes.

The results presented in Figure 2 showing the setting time of the geopolymer paste significantly varied with the type of alkali activator and ratio of FA/GGBS. On the activation of pastes with the alkali, i.e., NaOH + Na_2_SiO_3_ (CemN.C), the final setting time reduced, compared to the pastes activated with NaOH (Cem.C). It can be perceived from the results that the incorporation of Na_2_SiO_3_ increased the dissolution of solid material, i.e., FA and GGBS and accelerated the geopolymerization, thus contributing to the quick setting time [20,36]. Chindaprasirt, De Silva [37] explored in their study, that increasing Si/Al reduces the setting times. This also could be the reason of reduced setting time of CemN.C pastes compared to Cem.C pastes as silica supplied from Na_2_SiO_3_ increases the Si/Al ratio (as proved from EDS point analyses discussed in Section 3.6), leading to quick hardening of pastes.

The reduction in the final setting time is also observed with an increase in the GGBS content. The Cem.C paste containing 100% FA and 0% GGBS (F100S0) required a prolonged setting time of 2.16 h. This time tends to reduce as the amount of GGBS increases in the paste. Thus, with 0% FA and 100% GGBS (F0S100), the final setting time of the paste was observed to be 0.66 h. A similar trend was noted for CemN.C pastes, where the setting time of the F100S0 paste was 1.92 h and 0.42 h was noted for the F0S100 paste, which is comparatively lower than those for NaOH-activated pastes. The effects of increasing GGBS on decelerating setting times can be described in terms of increasing reactive Ca content from the GGBS source in the mixture, leading to the quick setting time [14]. Although FA contains 18.72% Ca was used to synthesis the pastes, based on this, the setting time values should have increased gradually for the pastes with increasing fly ash, but the results indicating the abrupt increase of setting time with increasing fly ash content. Hanjitsuwan, Hunpratub [9] suggests that the leaching of Ca content in the mixture reduces the setting time compared to the mixture where leaching of Ca was interrupted. This indicates that the Ca from fly ash was not/partially leached, but the Ca from the GGBS source leached completely; as a result, the setting time increased with increasing fly ash. The GGBS is more reactive than is fly ash, which was also observed in the previous studies [20,34]. Furthermore, the reactivity of fly ash accelerated when activated in NaOH + Na_2_SiO_3_ solution. As seen in the results, the fly ash-dominated paste, i.e., sample F100S0 when activated in NaOH + Na_2_SiO_3_, the setting time observed at 0.42 h., while for the same sample when activated in NaOH solution, the setting time prolonged to 0.24 h. and thus observed to be 0.66 h.

### 3.2. Compressive Strength

**Figure 3:**
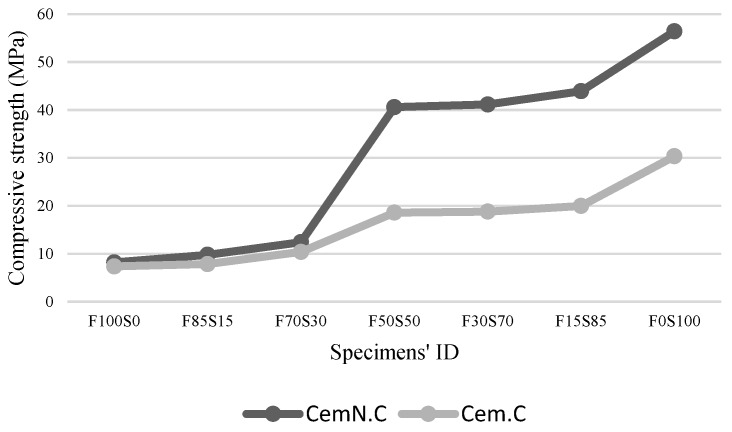
Compressive strength of Cem.C and CemN.C activated FA/GGBS samples.

The results of the compressive strength of FA and GGBS geopolymer mortar synthesized in two different alkalis (Cem.C and CemN.C) are presented in Figure 3. From the graphs, the compressive strength of the Cem.C samples is comparatively lower than that of CemN.C. This is due to the additional silica in the mixture supplied from Na_2_SiO_3_ source present in CemN.C samples as it increases SiO_2_ content in the pastes [4,5]. With its addition, the structure forms with close-packed anions, leading to the crossed-linked structure, and thus improves the compressive strength [5,38].

Furthermore, the increase in strength of both Cem.C and CemN.C samples was observed with the increase of GGBS content in the mixtures. As seen from Figure 3, on increasing the content of GGBS from 0% (F100S0) to 100% (F0S100), the strength increased from 7.35 MPa to 30.34 MPa for Cem.C samples and from 8.18 MPa to 56.43 MPa for CemN.C samples. A similar trend was observed in previous studies for class-F fly ash and GGBS blends, where strength increased with increasing GGBS content [19,20,21]. This was related to the increasing Ca in a mix with increasing GGBS content. Ismail, Bernal [39] discovered that the Ca rich pastes generally forms the C-S-H type gel, which produces the compact matrix and improves the strength. However, with the increase in Si and decrease in Ca, the C-S-H gel forms together with geopolymer gel, i.e., N-A-S-H type gel or form a hybrid C-N-A-S-H gel, which may result in strength loss. With the further increase of Si content and the presence of Ca content in a small quantity, the strength can reduce, as the N-A-S-H gel will be dominated in the structure where C-S-H or C-N-A-S-H type gel will no longer be observed. Based on this fact, the fly ash dominated samples obtained the reduced strength, but the strength should have reduced gradually with increasing fly ash content, since high Ca (18.72% Ca) fly ash was used in this study. As observed from the results in Figure 3, for Cem.C samples, when 15% fly ash was incorporated in GGBS, the strength reduced from 30.34 MPa (F0S100) to 19.96 MPa (F15S85) i.e., a 34.21% decrease was observed. Similarly, for CemN.C samples with the incorporation of 15% fly ash, the strength decreased from 56.43 MPa (F0S100) to 43.91 MPa (F15S85) i.e., a decrease of 22.19% was observed, which was lower than that of Cem.C samples. The abrupt decrease in strength indicated that the Ca in fly ash did not react in a matrix, which also was proved when the setting time prolonged with increasing fly ash, as discussed in Section 3.1 (final setting time). XRD results also evident in the unreactive Ca from fly ash source as it did not exhibit the traces of C-S-H as discussed in Section 3.4 (XRD analyses). Furthermore, the decrease percentile of CemN.C samples is lower than that of Cem.C samples indicating the reactivity of fly ash increased when activated in NaOH + Na_2_SiO_3_. When the FA completely replaced by GGBS, the highest compressive strength value was observed, i.e., 30.34 MPa and 56.43 MPa for both Cem.C and CemN.C samples respectively, which denotes the increase in reactive Ca with increasing GGBS content leading to more compact matrix.

### 3.3. Volume of Impervious Portion, Apparent Porosity and Water Absorption

**Figure 4:**
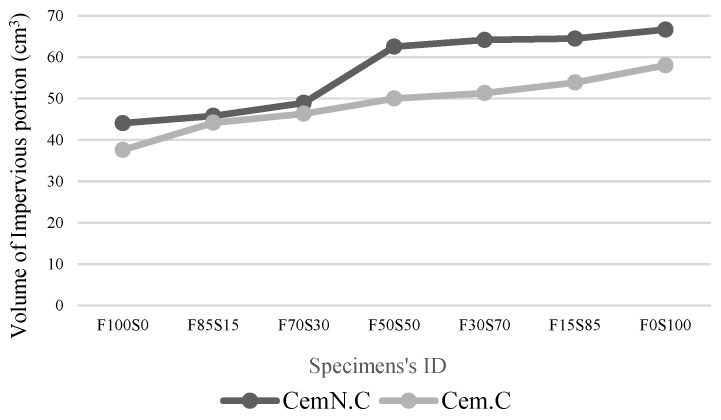
Volume of impervious portion of Cem.C and CemN.C activated FA/GGBS samples.

**Figure 5:**
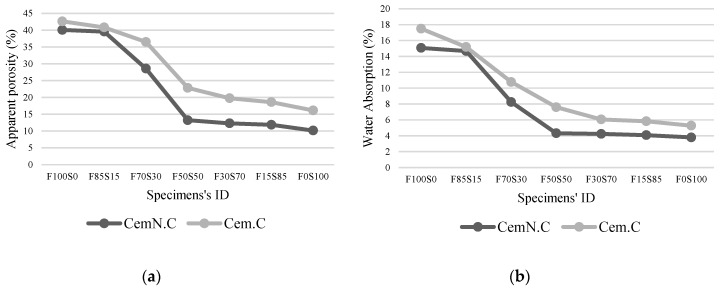
Cem.C and CemN.C activated FA/GGBS samples: (**a**) apparent porosity; (**b**) water absorption.

Figure 4 and Figure 5a,b show the impervious portion, apparent porosity, and water absorption respectively of samples of various FA to GGBS ratios of both Cem.C and CemN.C series. As seen in Figure 4, the CemN.C samples comparatively obtained the highest impervious portion (i.e., 44.05 cm^3^ with 0% GGBS and 66.66 cm^3^ with 100% GGBS) than Cem.C samples (i.e., 37.6 cm^3^ with 0% GGBS and 58.025 cm^3^ with 100% GGBS) indicating CemN.C sample are more impervious than Cem.C samples. Results revealed the impervious portion is in agreement with the result of apparent porosity, Figure 5a and water absorption, Figure 5b where the CemN.C series compared to the Cem.C obtained the low porosity (i.e., 40.05% with 0% GGBS and 10.14% with 100% GGBS) and low water absorption (i.e., 15.07% with 0% GGBS and 3.8% with 100% GGBS). For the Cem.C series the porosity and water absorption observed with 0% GGBS is 42.62% and 17.49%, respectively and the porosity and water absorption with 100% GGBS observed was 16.12% and 5.28%, respectively. This confirms that the addition of Na_2_SiO_3_ increased the soluble silicates within the matrix, which improved the inter-particle bonding and made the matrix less porous, resulting in improved strength compared to NaOH [40]. The results in Figure 4 show the increasing trend in volume of impervious portion as the content of GGBS increased from 0% to 100%. The trend shown in the impervious portion was the inverse of the apparent porosity of Figure 5a and water absorption, and Figure 5b indicated the decrease in open pores with the increasing GGBS content, which prevented the water from penetration.

**Figure 6:**
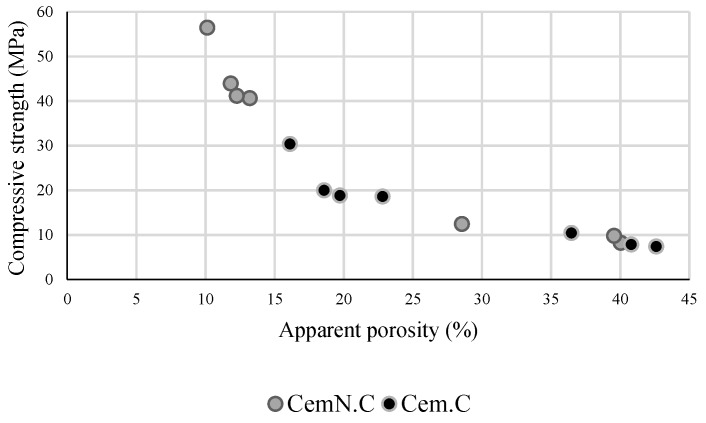
Relationship between apparent porosity and compressive strength of Cem.C and CemN.C activated FA/GGBS samples.

The results obtained from the Archimedes principles supports the values obtained from the compressive strength test. As illustrated quantitatively in Figure 6, the compressive strength reduced with the increasing porosity, which indicated the strong relationship between porosity of the matrix and the compressive strength regardless of alkali type and chemical composition of matrix. For instance, the Cem.C-F100S0 obtained higher porosity (42.62%) indicating the development of a loose matrix leading to the lowest compressive strength (7.37 MPa). While CemN.C F100S0 obtained comparatively low porosity (40.05%) and improved compressive strength (8.18 MPa).

### 3.4. XRD Analysis

**Figure 7:**
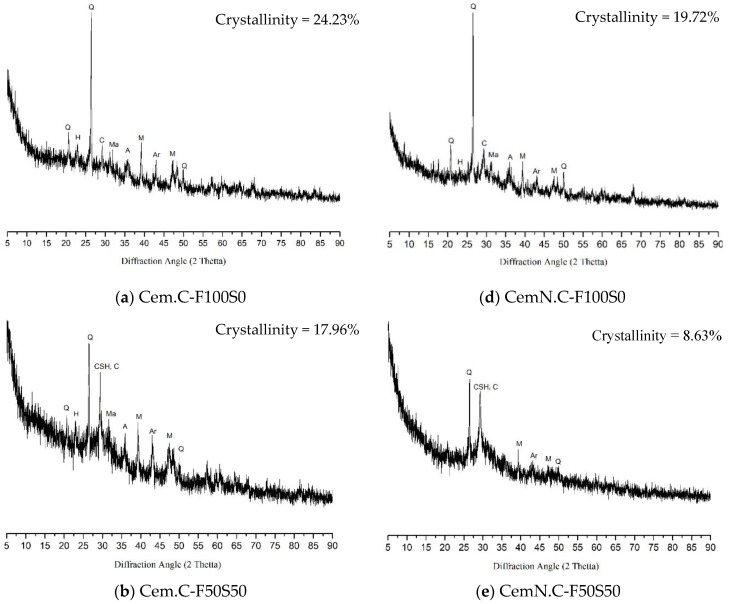

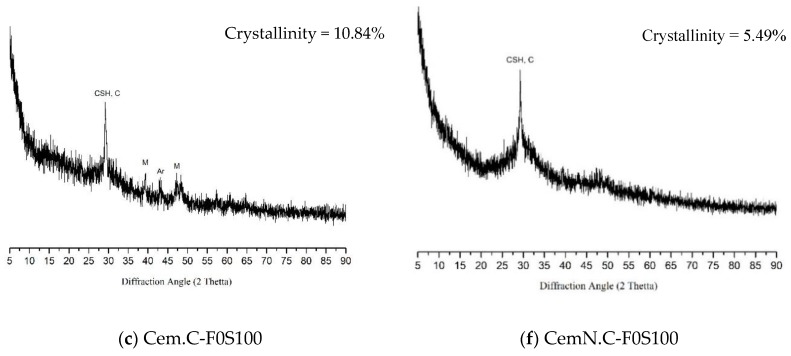
XRD patterns of Cem.C activated FA/GGBS: (**a**) 100/0, (**b**) 50/50, (**c**) 0/100 and CemN.C activated FA/GGBS: (**d**) 100/0, (**e**) 50/50, (**f**) 0/100 pastes. Note: Q = quartz (00-046-1045 > SiO_2_), Ma = magnetite (01-079-0418 > Fe_3_O_4_), M = mullite (00-015-0776 > Al_6_Si_2_O_13_), CSH = calcium silicate hydrate (00-22-0600 > 2CaSiO_3_.3H_2_O), C = calcite (01-071-3699 > CaCO_3_), Ar = aragonite (01-076-0606> CaCO_3_), H = hydrosodalite (00-011-0401 > Na_4_Al_3_Si_3_O_12_(OH)), A = albite (00-009-0466 > NaAlSi_3_O_8_).

Figure 7 shows the XRD pattern of NaOH (Cem.C) and NaOH + Na_2_SiO_3_ (CemN.C) activated F100S0, F50S50 and F0S100 FA/GGBS composites. It is clear from the XRD patterns that the intensity of peaks varied with the type of activator, as well as the ratio of FA/GGBS. The sample F100S0, from both Cem.C series, Figure 7a and CemN.C series Figure 7d, was composed of the crystalline phases of quarts, magnetite, mullite, arghonite, calcite, albite, and hydrosodalite, indicating the elements of fly ash were partially in a crystalline form. The intensity of these peaks (except quartz) slightly reduced when fly ash was activated with a NaOH + Na_2_SiO_3_ alkali solution (CemN.C series). Accordingly, the percentage of crystallinity of Cem.C F100S0 was 24.23%, which reduced to 19.72% for CemN.C. The formation of hydrosodalite (form of zeolites) was observed in both NaOH and NaOH + Na_2_SiO_3_ activated fly ash, due to the presence of high SiO_4_ and AlO_4_ content than CaO content in a composite [41,42]. However, the intensity of hydrosodalite peak was stronger in the Cem.C series, which resulted in low strength compared to that of the CemN.C series, which was due to the presence of amorphous silicate supplied from Na_2_SiO_3_ [4]. Additionally, the formation of calcite peaks around 2θ = 28° in both CemN.C and Cem.C F100S0 indicated the presence of Ca in fly ash. However, the Ca in fly ash remained unreactive as it did not contribute to the C-S-H formation [4,43,44]. Furthermore, Cem.C F100S0 led to the formation of a low-intensity hump between 2θ = 18°–35°, while CemN.C F100S0 led to the formation of a high-intensity hump between 2θ = 23°–38°. This could be associated with the formation of an amorphous compound in gel due to the presence of amorphous silica (in the form of Na_2_SiO_3_) added to the paste.

With the incorporation of the GGBS, the reduction in the intensity of the crystalline peaks, as well as in percentage of crystallinity was observed in the F50S50 pattern of both Cem.C, Figure 7b and CemN.C, Figure 7e samples. The Cem.C F50S50 was composed of the same crystal phases as those of Cem.C F100S0 but with reduced intensity and with 17.96% crystallinity. It is worth observing that the calcite peak around 2θ = 28° showed traces of C-S-H gel, indicating the formation of C-S-H gel with the addition of reactive Ca from GGBS source. Additionally, with the incorporation of GGBS, the intensity of the hydrosodalite peak reduced. This was caused by the increase in CaO content in the mix as the fly ash was partially replaced by the GGBS. The reduction of hydrosodalite peak intensity was a clear indication of the development of N-A-S-H gel, which is responsible for improved strength. Phoo-ngernkham, Maegawa [4] and Qiu, Zhao [20] also showed the similar trend for a sample consisting of class-F FA /GGBS in a 50/50 ratio. Comparatively, the pattern of CemN.C F50S50, Figure 7e, exhibited the peaks corresponding to quartz, calcite with traces of C-S-H, mullites, and arghonite, but with reduced intensity and reduced crystallinity, i.e., 8.63%. No formation of hydrosodalite was observed in this pattern; thus, the strength improved compared to that of Cem.C F50S50. This was mainly due to the coexistence of C-S-H and N-A-S-H gel [39]. Apart from the crystalline peaks, the intensity of the hump also increased for the samples F50S50 compared to those for F100S0, indicating the increase in the formation of amorphous phases supplied from the GGBS source. However, hump intensity of the sample Cem.C F50S50 was comparatively lower than that of CemN.C F50S50, as observed at 2θ = 25°–38°.

For sample Cem.C F0S100, as shown in Figure 7c, the XRD pattern was mainly composed of amorphous phases as it is clear from the reduced crystallinity percentage, i.e., from 17.96% to 10.84% with fewer diffraction peaks of mullite, aragonite, and calcite with traces of CSH gel. The intensity of calcite, C-S-H, peak around 2θ = 29° was comparatively stronger than those seen in the F50S50 pattern, indicating the increase in CaO, leading to the formation of calcite and C-S-H gel, which was solely responsible for the reduced setting time to 0.66 h, increased strength of 30.34 MPa, and reduction of porosity to 16.12% [4,20]. CemN.C F0S100, Figure 7f, on the other hand, showed the lowest crystallinity of 5.49% with only crystalline peaks of calcite and C-S-H gel. The broad hump at 2θ = 23.5°–38° indicates the main composition of amorphous phases incorporated from both GGBS and Na_2_SiO_3_, leading to the lowest setting time of 0.42 h, the highest compressive strength of 56.43 MPa, and lowest porosity of 10.14%.

### 3.5. SEM Micrographs

**Figure 8:**
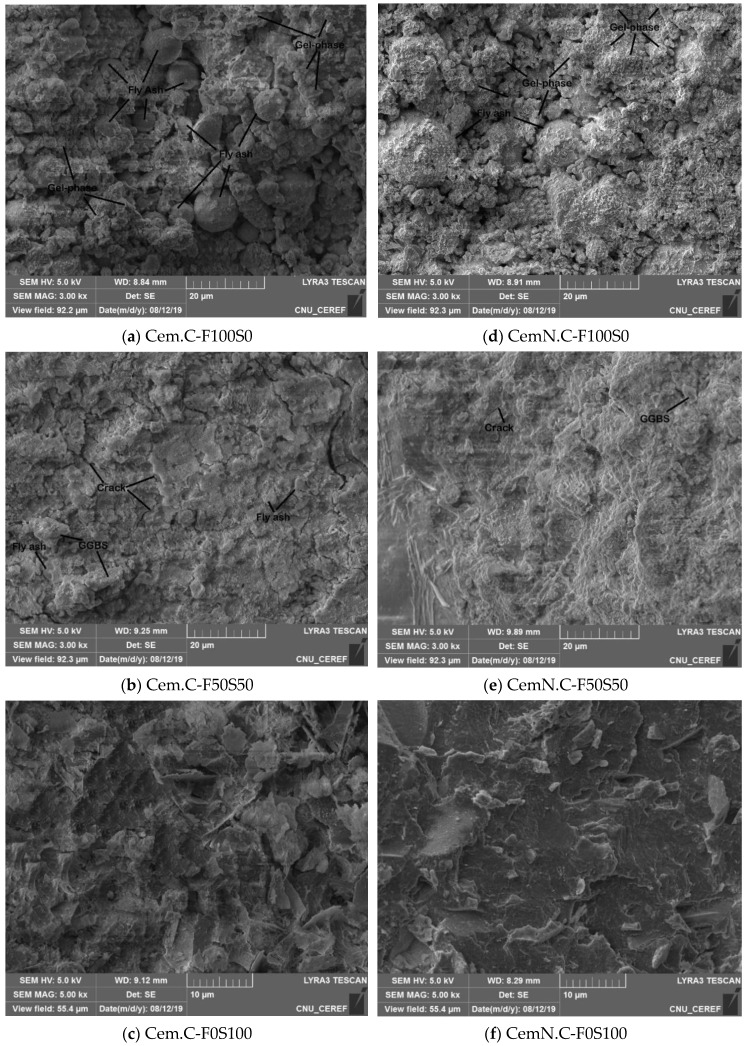
SEM images of Cem.C activated FA/GGBS: (**a**) 100/0, (**b**) 50/50, (**c**) 0/100 and CemN.C activated FA/GGBS: (**d**) 100/0, (**e**) 50/50, (**f**) 0/100 pastes.

**Figure 9:**
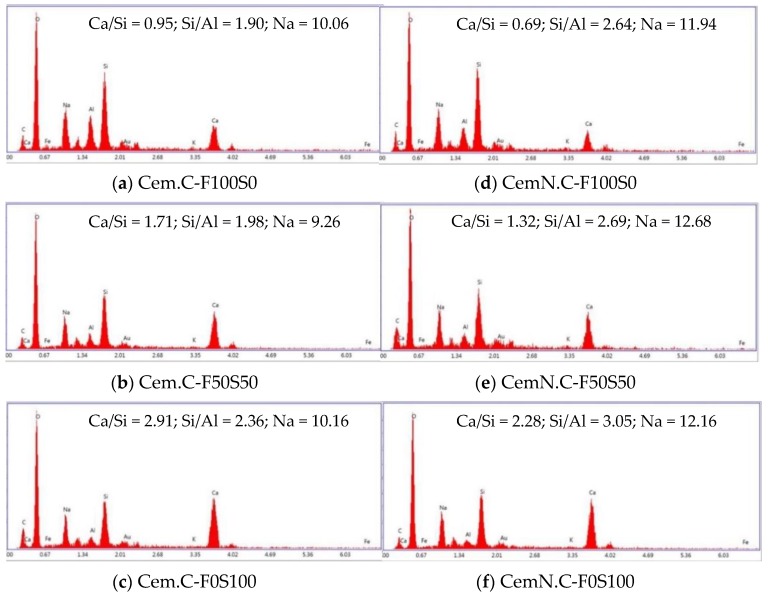
EDS analyses of Cem.C activated FA/GGBS: (**a**) 100/0, (**b**) 50/50, (**c**) 0/100 and CemN.C activated FA/GGBS: (**d**) 100/0, (**e**) 50/50, (**f**) 0/100 pastes.

SEM analyses of NaOH and NaOH + Na_2_SiO_3_ activated F100S0, F50S50 and F0S100 FA/GGBS composites is shown in Figure 8. When compared the micrographs of sample Cem.C F100S0 in Figure 8a, with CemN.C F100S0 in Figure 8d, it can be seen that the fly ash remained undissolved and some of the particles are surrounded by the gel phases, thus resulting in a lower strength of 7.35 MPa. While the sample CemN.C F100S0 in Figure 8d, the fly ash particles are partially dissolved and the matrix shows the maximum gel phases, in which the particles are embedded, results in slight gain in compressive strength of 8.18 MPa. This confirms that the fly ash starts to dissolve when activated in NaOH + Na_2_SiO_3_, which leads to the leaching of the elements such as Si, Al, and Ca into the matrix, as a result, the strength and the homogeneity of matrix improved. Additionally, the formation of more gel phases in sample CemN.C F100S0 indicates the acceleration in geopolymerization with the incorporation of Na_2_SiO_3_ [4]. The sample F50S50 as shown in Figure 8b,e compared to samples F100S0 Figure 8a,d exhibits denser matrix, due to the increase of GGBS and decrease of fly ash content in the paste. Whereas, the alkali type also showed significant effect on the matrix, as the composite activated in NaOH as shown in Figure 8b is less dense than the composite activated in NaOH + Na_2_SiO_3_ in Figure 9e. It supports the results revealed by the above experimental results showing the more strength and less porosity for the samples activated with NaOH + Na_2_SiO_3_. Further increase of GGBS to 100% and decrease of FA to 0% in samples F0S100 made the matrix more homogeneous and compact as shown in Figure 8c,f. Comparatively, increased homogeneity in the matrix can be observed in sample CemN.C F0S100 in Figure 8f due to the additional silicate from Na_2_SiO_3,_ which accelerate the gel formation in matrix.

In conclusion, as seen through images (a)–(f) in Figure 8, the reduction in the amount of fly ash leads to a homogeneous and denser matrix due to less or no reactivity of the fly ash. It supports the results revealing the increasing strength with the reduction of fly ash content. Meanwhile, the loose matrix in the higher fly ash content samples causes porosity in the matrix, which was proved in the above study, i.e., Figure 5a. Similar trends were found in the previous studies, where increasing fly ash leads to the porous matrix [4,20,45]. The alkali type also showed significant effects on the structure of the matrix, as particles become more reactive when activated in NaOH + Na_2_SiO_3_ leading to the homogeneous and compact matrix, resulting in reduced porosity and improved compressive strength.

Note: The SEM micrographs exhibit micro-cracks—these cracks were observed in almost all the micrographs collected for the mentioned samples. It is a result of excessive load applied on the sample to obtain the mini samples for SEM analyses.

### 3.6. SEM-EDS Point Analyses

In EDS point analyses, the spectrum was collected from three different areas of each sample to obtain the average elemental composition, which is used to derive the ratios as shown in Figure 9. The spectrum graphs show the increase in silica and the slight increase in Na for the samples activated in NaOH + Na_2_SiO_3,_ i.e., the CemN.C series in Figure 9d–f. It confirms that the silica was supplied to the paste with the addition of Na_2_SiO_3_ as a result, the gel, i.e., C-S-H and/or geopolymer gel formed with close-packed anions resulting in a strength gain [5,38]. Additionally, the spectrum showed the increasing Si/Al ratio for CemN.C samples, which was also associated with the reduced setting time as discussed in Section 3.1. Increases in the Si/Al ratio was also observed for the samples with increasing GGBS, with which Al content reduced. It led to the formation of Si-O-Si bonds which were stronger than Si-O-Al bonds resulting in increased strength [46]. The increase in the intensity of Ca bar was observed on the samples with increasing GGBS in the matrix, which led to the increased Ca/Si ratio. This suggests that the highest Ca/Si ratio promoted the C-S-H bond formation, which filled the open voids resulting in reduced porosity, and increased strength [47]. However, the Ca/Si ratio for samples activated with NaOH + Na_2_SiO_3_ (CemN.C), as shown in Figure 9d–f, was comparatively less, but the strength was higher than NaOH (Cem.C) activated samples, Figure 9a–c. This was due to the supplied amorphous silica from Na_2_SiO_3_ source, which reduced the Ca/Si ratio but the increase in amorphous silica contributed in creating the compact anions as explored by Fernández-Jiménez and Puertas [27] in their study.

## 4. Conclusions

This paper presents the extensive study on the effects of NaOH and NaOH + Na_2_SiO_3_ solutions and the incorporation of class-C fly ash on the setting time, strength, porosity, crystallinity, and microstructure of FA and GGBS blends. Based on analyses and discussion the following conclusions can be drawn.

1. The alkaline activator plays an important role in developing a stronger and denser matrix. As explored, addition of Na_2_SiO_3_ was one of the parameters for improving strength and microstructure as it accelerated geopolymerization by supplying the amorphous silica from Na_2_SiO_3_ source. This favors the formation of cross-linked structures in the matrix, which is responsible for strength development. The study also examined the improved reactivity of fly ash when activated in NaOH + Na_2_SiO_3,_ which also contributed to the strength development and improvement in homogeneity of the matrix.
2. The other factor effecting the strength and microstructure of FA/GGBS blends is the reactivity of raw materials. Class-C fly ash, when activated, remained unreactive/partially reactive, which limits the leaching of major element such as Si, Al and Ca to the matrix. The limit in the leaching of Ca was initially observed when the setting time increased abruptly with the increasing fly ash content in the sample. Accordingly, XRD analyses reveals the Ca in the fly ash did not contribute in forming C-S-H bonds but the formation of CSH gel was dependent on the reactive Ca from the GGBS source, which resulted in the increased strength, reduced porosity and compact matrix. SEM micrographs further clarified the less reactivity of fly ash in a matrix.
3. The influence of NaOH and NaOH + Na_2_SiO_3_ alkali solutions in class-C FA and GGBS is explored in this study where partial reactivity of fly ash in NaOH + Na_2_SiO_3_ solution was observed. Based on this study, it is hypothesized that the leaching of Ca from class-C fly ash can be possible if activated in a high concentrated alkali solution. Therefore, the reactivity of class-C fly ash and GGBS in different alkaline solutions with different concentration should be explored in future studies.

## Figures and Tables

**Table 1:** Nominal chemical composition of fly ash (FA) and ground granulated blast-furnace slag (GGBS).

**Elements (Wt. %)**	**O**	**Fe**	**Na**	**AL**	**Si**	**Au**	**S**	**Cl**	**K**	**Ca**
FA	43.44	2.01	0.65	10.33	18.16	2.38	1.50	0.82	1.53	18.72
GGBS	35.43	0.51	0.47	7.37	14.57	1.38	-	-	0.35	39.92
**Oxide (Wt. %)**	**SiO_2_**		**Al_2_O_3_**		**CaO**	**Fe_2_O_3_**		**Na_2_O**		**K_2_O**
FA	38.84		19.52		26.19	2.87		0.87		1.84
GGBS	31.17		13.92		55.85	0.729		0.63		0.421

**Table 2:** Mixture proportion of FA and GGBS pastes activated in NaOH and NaOH + Na_2_SiO_3_.

Specimen ID	FA (wt.%)	GGBS (wt.%)	Alkali Activator	Al/S	W/Mix
NaOH activated pastes (Cem.C)					
F100S0	100	0	NAOH	0.4	0.075
F85S15	85	15			0.077
F70S30	70	30			0.079
F50S50	50	50			0.087
F30S70	30	70			0.091
F15S85	15	85			0.098
F0S100	0	100			0.1
NaOH + Na_2_SiO_3_ activated pastes (CemN.C)					
F100S0	100	0	NaOH+ Na_2_SiO_3_	0.4	0.104
F85S15	85	15			0.107
F70S30	70	30			0.112
F50S50	50	50			0.123
F30S70	30	70			0.129
F15S85	15	85			0.132
F0S100	0	100			0.138
